# Urban structure reinforces attitudes towards tsunami evacuation

**DOI:** 10.1038/s41598-023-33562-9

**Published:** 2023-05-17

**Authors:** Fumiyasu Makinoshima, Yusuke Oishi, Fumihiko Imamura

**Affiliations:** 1 Fujitsu Limited, Kawasaki, 211-8588 Japan; 2 International Research Institute of Disaster Science, Tohoku University, Sendai, 980-8572 Japan

**Keywords:** Natural hazards, Civil engineering

## Abstract

Evacuation is a critical life-saving action, especially in devastating natural hazards such as near-field tsunamis. However, the development of effective evacuation measures remains challenging to the extent that a successful example has been referred to as a ‘miracle’. Here we show that urban structures have the potential to reinforce attitudes towards evacuation and significantly influence the success of tsunami evacuation. Agent-based evacuation simulations revealed that a distinctive root-like urban structure formed in ria coasts reinforces positive evacuation attitudes by effectively gathering evacuation flows and leads to higher evacuation rates compared to typical grid-like urban structures, which can explain the regional differences in the number of casualties in the 2011 Tohoku tsunami. Although a grid-like structure reinforces negative attitudes under low evacuation tendencies, with leading evacuees, its dense feature helps to propagate positive attitudes and drastically improve evacuation tendencies. These findings pave the way for making successful evacuation inevitable through harmonised urban and evacuation plannings.

## Introduction

Evacuation is a simple, yet critical behaviour that greatly reduces casualties during devastating natural disasters. Particularly in near-field tsunamis, for which a short lead time for evacuation is available, the importance of prompt evacuation has been recognised and discussed on the basis of previous experiences^1^; however, implementing a successful evacuation is challenging despite its behavioural simplicity. In the 2004 Indian Ocean tsunami, we experienced the tremendous loss of life reaching nearly 230,000. Although this severe human loss was explained by the lack of tsunami preparedness programmes in the affected countries^2^, the immense human loss of nearly 20,000 in the following 2011 Tohoku tsunami again occurred even with the advanced warning system and preparedness efforts^3^. Recent advances in tsunami observation networks^2,4–6^ and real-time tsunami forecasting techniques^7–12^ especially after the 2011 Tohoku event, help provide richer hazard information and enable the issuance of rapid and accurate early warning to prompt evacuations. Nevertheless, these historical events suggest that successful evacuation cannot be achieved solely through these technological developments. A long-standing open question has been how to greatly motivate evacuation behaviours towards zero casualty—the ultimate goal of tsunami disaster risk reduction efforts.

Evacuation ultimately depends on individual decision-making; hence, researchers and practitioners have been putting emphasis largely on individual or community efforts, such as traditions^13–15^, education^16–19^, and drills^20–22^, to encourage awareness and preparedness for future tsunami evacuations. Although there is no doubt that these measures improve individual evacuation capability and awareness, the measures have generally been reaching only some of the members of society^21,23^, limiting their effect. Some studies suggest that improved awareness does not necessarily lead to behavioural changes^24^. Since the actual evacuation behaviour relies largely on individual risk perception and knowledge^25^, when a hazard strikes, the success of these measures always suffers from negative psychological biases associated with the evacuation process^26,27^ and fading awareness^23,28^, which make evacuation success uncertain. As a result, developing effective evacuation measures towards zero casualty remains challenging to the extent that people call a successful example ‘miracle’^29^.

Here we show that urban structures have the potential to control evacuation flows and reinforce attitudes towards evacuation, which leads to the development of novel evacuation measures. An agent-based evacuation simulation applied to two cities that have different urban structures in Tohoku, Japan reveals a significant difference in evacuation tendencies between the cities, even with similar evacuation attitudes. Detailed analyses of the simulation unravel the mechanism of exciting different evacuation tendencies, i.e., the attitude reinforcement mechanism inherent in an urban structure, which can explain the regional differences in the number of casualties of the 2011 Tohoku tsunami. Further simulations demonstrate that the effect of a small number of leading evacuees can be greatly enhanced with the uncovered attitude reinforcement mechanism, leading to a significant improvement in evacuation tendencies. These findings suggest that successful evacuations can be designed systematically and do not require a miracle.

## Results

### Different evacuation tendencies between cities

**Figure 1:**
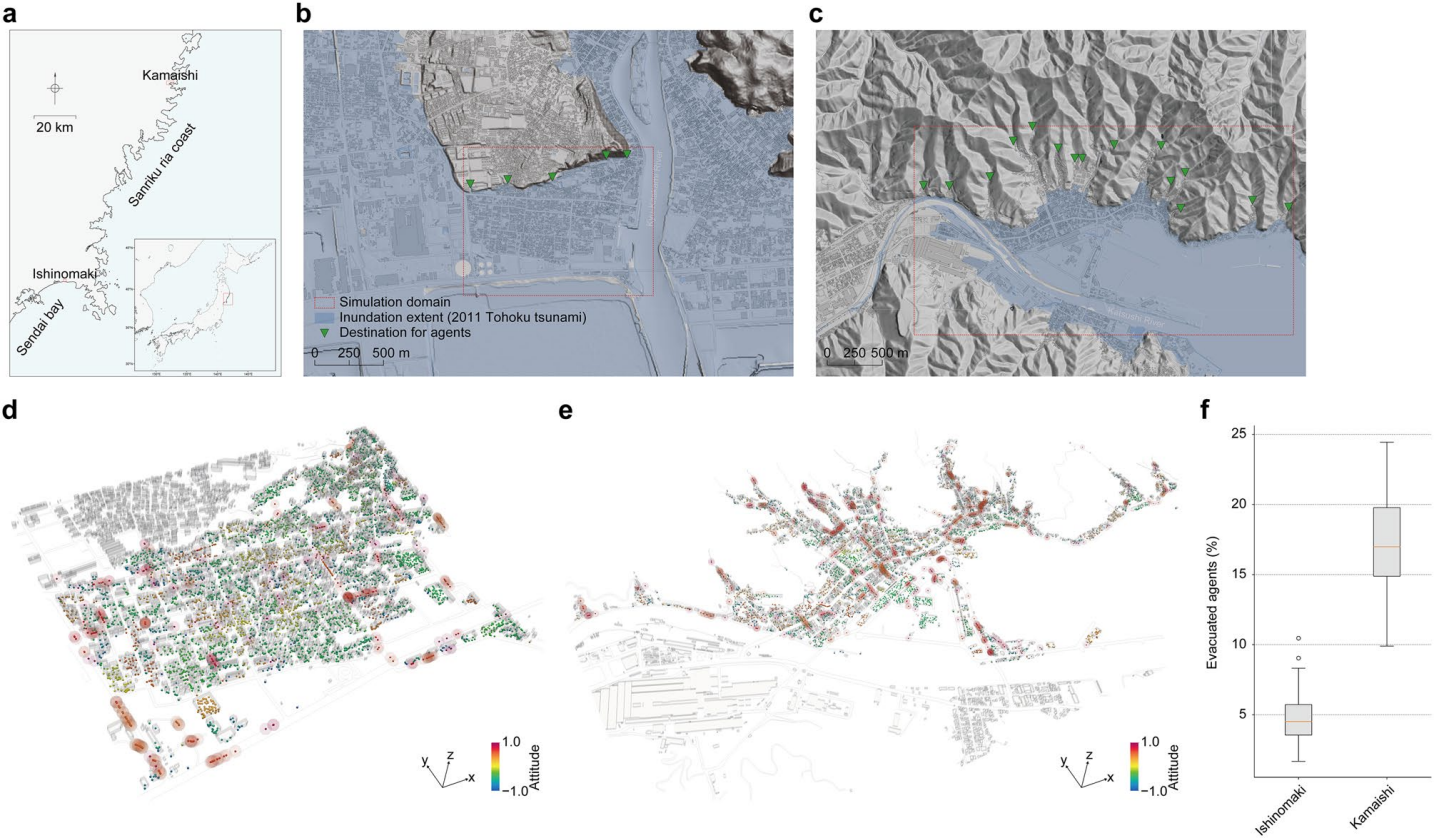
Simulation domains and results of agent-based tsunami evacuation simulation. (**a**) Simulation domains presented in a large-scale map. Simulation domain for Ishinomaki is in a plain area located in the northern part of Sendai bay. Simulation domain for Kamaishi is located within Sanriku ria coast. (**b**) Simulation domain for Isinomaki in a small-scale map. Typical grid-like urban structure was formed in the plain area where a large extent of the tsunami inundation was experienced due to its low-lying flat feature. (**c**) Simulation domain for Kamaishi in a small-scale map. The distinctive root-like urban structure was naturally formed along with the topography of ria coast. (**d**, **e**) Snapshots of the evacuation simulation ($t=60~s$) for Ishinomaki and Kamaishi, respectively. The evacuating agents are highlighted with larger spheres indicating their communication extent. More active evacuation behaviours (coloured in red) are found in Kamaishi compared to those in Ishinomaki where many non-active agents (coloured in green to yellow) can be observed. (**f**) Comparison of the evacuation completion ratios. Even with similar evacuation attitudes, Kamaishi achieved a higher evacuation completion. The box plots consist of the median line, box limits (inter-quartile range), whiskers (1.5 $\times$ inter-quartile range from upper or lower quartiles) and dots (fliers larger or smaller than whiskers). Each box plot includes $n=120$ simulation runs. Figures were made with the Generic Mapping Tools^34^ (https://www.generic-mapping-tools.org/), QGIS^35^ (https://qgis.org/) and ParaView^36^ (https://www.paraview.org/).

We applied an agent-based on-foot evacuation simulation^30^ to the coastal areas of Ishinomaki and Kamaishi cities, which experienced the devastating 2011 Tohoku tsunami. In this model, agents have their own attitudes towards evacuation for their decision-making, which can be updated through communications with surrounding agents. The model can simulate individual comprehensive evacuation processes, i.e., decision-making based on attitudes towards evacuation, communications with surrounding agents to update their attitudes and detailed physical evacuation movements. The model can be considered a simplified numerical implementation of theoretical decision-making models often used to explain evacuation behaviours^31–33^ (see Methods for details of the model). The simulation domain for Ishinomaki is located at the northern part of Sendai bay (Fig. 1a) and has a grid-like urban structure often seen in typical urban areas (Fig. 1b). The simulation domain for Kamaishi is situated at Sanriku ria coast (Fig. 1a) and has a distinctive root-like urban structure that is naturally formed along the funnel-shaped topography of a ria coast (Fig. 1c). These simulation domains were resolved with a $1 \times 1$ $\textrm{m}^2$ mesh.

Based on existing investigations, we considered randomly distributed 5000 agents both in Ishinomaki and Kamaishi. Since this paper focuses on investigating the evacuation behaviours in different urban structures rather than reproducing the actual behaviours, we consistently assumed that agents’ attitudes towards evacuation follow a uniform random distribution between $-1.0$ and 1.0 in both areas (see Methods for details of the simulation). The population and their evacuation attitudes were controlled; therefore, the primal difference between these simulations is their urban structure, such as building density and road network. With these inputs, we conducted the simulations and observed the behaviours of agents, such as the change in evacuation attitudes and behaviours.

Even with the same population and attitudes towards tsunami evacuation, the simulation results showed more active evacuation behaviours in Kamaishi compared to that in Ishinomaki (Fig. 1d,e). Consequently, a clear difference in the evacuation completion ratio for the two cities was confirmed (Fig. 1f). Here, stochastic simulations with 120 simulation runs were conducted to evaluate the uncertainty in the initial attitudes distribution; the result showed a significant difference, reaching 263% of the relative difference on average. This result suggests that various evacuation tendencies in different regions during the 2011 Tohoku tsunami were not caused only by different attitudes towards evacuation.

### Attitude reinforcement mechanism inherent in urban structures

**Figure 2:**
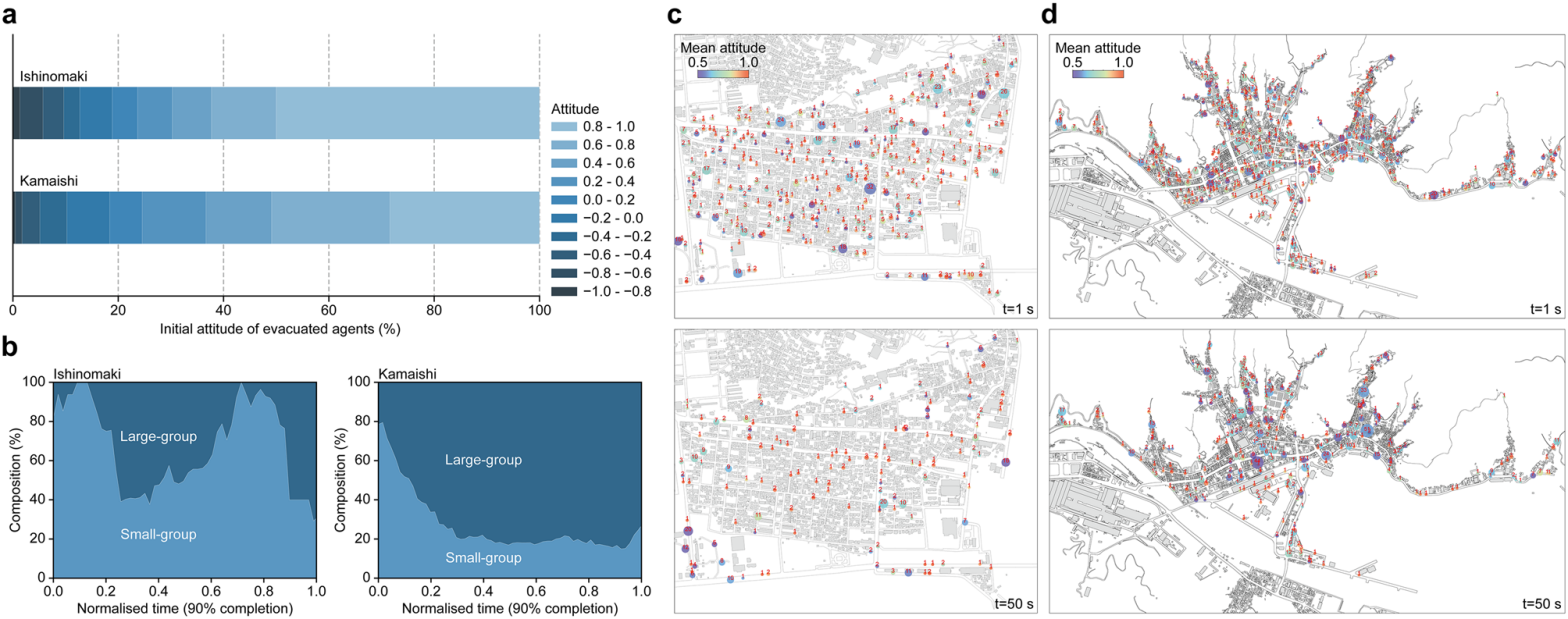
Different evacuation behaviours found in different cities. (**a**) Comparison of the initial attitude distributions of evacuated agents. Agents with relatively lower evacuation attitudes could evacuate in Kamaishi compared to those in Ishinomaki. (**b**) Comparisons of the timeseries of the evacuating cluster size. The number of agents in clusters of 10 or more agents was counted as large-groups. The time is normalised for comparison considering different evacuation distances in different cities. (**c**, **d**) Evolution of clusters of the agents with positive attitudes in Ishinomaki and Kamaishi, respectively. The centres of the clusters are plotted with the number of agents in the clusters. The size of the plots corresponds to the cluster size. While small-group evacuees with high evacuation attitudes are consistently observed in Ishinomaki, large-group evacuees with relatively lower evacuation attitudes are likely to be observed in Kamaishi. Maps were made with GeoPandas^37^ v.0.11.1 (https://geopandas.org/).

We analysed the median simulation cases to investigate the mechanism generating the significant difference in evacuation tendencies. A difference in the attitude distribution of evacuated agents between two cities can be confirmed (Fig. 2a). The comparison of the initial attitude of evacuated agents reveals that more agents with relatively lower evacuation attitudes ($0.2 \le A_i \le 0.8$) could complete their evacuation in Kamaishi (47.0%) compared with only 26.3% in Ishinomaki. An analysis of the size of the clusters of closely communicating evacuees further shows a clear difference (Fig. 2b). Here, we analysed the communication network to extract the clusters (see Methods for details of network analyses) and counted the number of evacuating agents in clusters of 10 or more agents as large-groups, and otherwise small-groups. While the small-group evacuation is dominant in Ishinomaki ($\sim$ 40 to 100%), the stably growing composition of the large-group evacuation in Kamaishi reaches $\sim 80\%$. Simulation snapshots show that these small-group evacuees in Ishinomaki have high positive attitudes, i.e., $A_i >\sim 0.8$, whereas the large-group evacuees in Kamaishi have positive but relatively lower evacuation attitudes, i.e., $A_i <\sim 0.6$, (Fig. 2c,d). These analyses show that the large-group evacuations excited especially in Kamaishi, involving many agents with moderately positive attitudes, are the main cause of the significant difference found in the evacuation completion.

**Figure 3:**
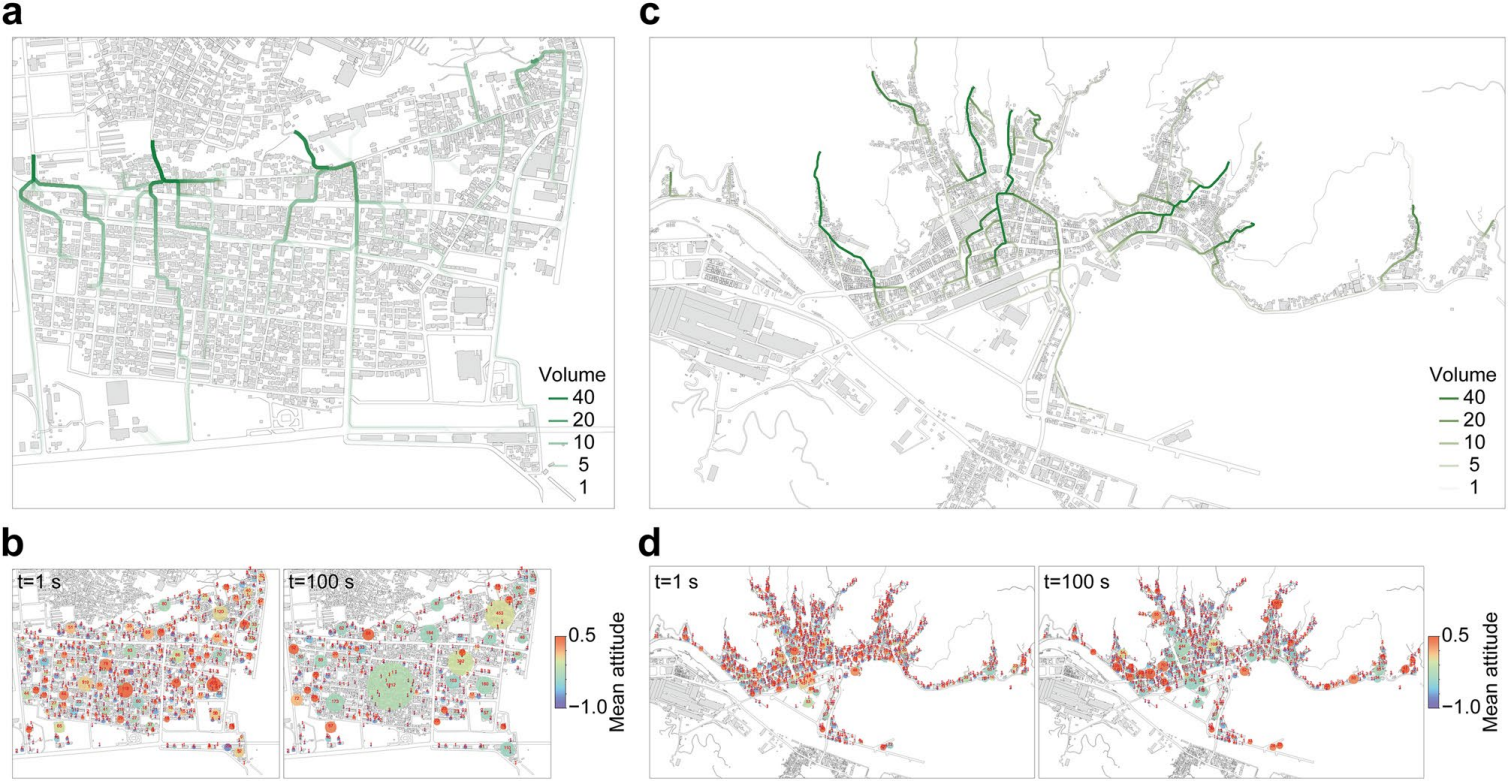
Attitude reinforcement mechanisms inherent in urban structures. (**a**) Trajectories of evacuated agents in Ishinomaki. The grid-like structure causes dispersed evacuation flows and makes them difficult to merge. (**b**) Evolution of clusters of the agents with negative attitudes in Ishinomaki. The centres of the clusters are plotted with the number of agents in the clusters. The size of the plots corresponds to the cluster size. The dense grid-like structure helps the growth of clusters with negative attitudes and results in the formation of large clusters with negative attitudes that negatively influence the evacuation of agents with positive attitudes. (**c**) Trajectories of evacuated agents in Kamaishi. The distinctive root-like structure effectively gathers evacuation flows and helps them form strong clusters with positive attitudes that cannot be influenced by clusters with negative attitudes. (**d**) Evolution of clusters of the agents with negative attitudes in Kamaishi. The centres of the clusters are plotted with the number of agents in the clusters. The size of the plots corresponds to the cluster size. The relatively sparse root-like structure limits the growth of clusters with negative attitudes. Maps were made with GeoPandas^37^ v.0.11.1 (https://geopandas.org/).

The urban structure causes this difference in evacuation behaviours. The grid-like urban structure in Ishinomaki makes evacuation flows include more components perpendicular to the coast rather than those parallel to the coast (Fig. 3a). As a result, the evacuation flows are dispersed and merge immediately before the roads heading towards higher grounds. The grid-like structure also has a dense urban structure that accelerates the formation of clusters of agents with negative attitudes, resulting in large clusters with several hundreds of agents with negative attitudes (Fig. 3b). These large clusters negatively influence the evacuation of agents with moderately positive attitudes before they form strong clusters with positive attitudes. The combined effect of dispersed evacuation flows and the growth of clusters with negative attitudes caused by the dense grid-like structure prevents evacuees with moderately positive attitudes from forming clusters, causing depressed evacuation tendencies. Consequently, the small-group evacuation with high evacuation attitudes becomes dominant in Ishinomaki.

In contrast, the root-like urban structure in Kamaishi effectively gathers evacuation flows (Fig. 3c). This structural feature enables evacuees to form clusters with positive attitudes even in the early phase of their evacuation processes. The evacuation attitude of these clusters is moderately positive, but they are less likely to be affected by clusters with negative attitudes since their attitudes are maintained through close communications within the clusters. Additionally, the city blocks in Kamaishi are separated by wider roads, making a sparser urban structure than that in Ishinomaki. The sparser structure prevents clusters with negative attitudes from becoming large, thereby limiting their size (Fig. 3d). As a result, rapidly formed clusters with positive attitudes are not influenced by the relatively small clusters with negative attitudes and can complete their evacuation movements towards higher grounds, leading to a higher evacuation completion ratio.

### Significantly improved evacuation tendency by leading evacuees

**Figure 4:**
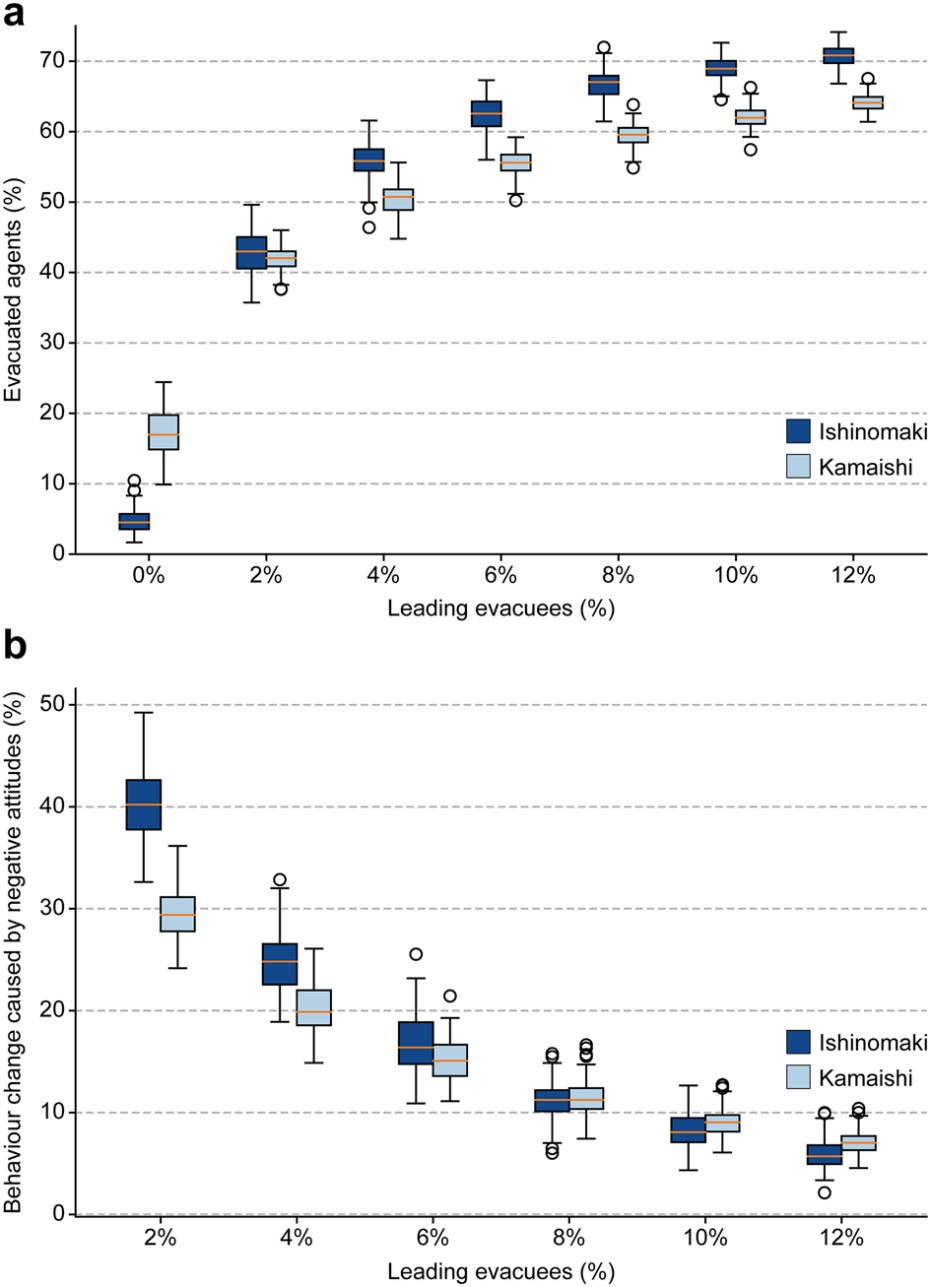
Significant improvement in evacuation tendencies with leading evacuees. (**a**) Evacuation completion ratios with different numbers of leading evacuees. Attitude reinforcement mechanism helps to effectively propagate positive attitudes from a small number of leading evacuees and improves the evacuation tendencies. (**b**) Ratios of behaviour change caused by negative attitudes during evacuation under varying numbers of leading evacuees. A low probability of being influenced by negative attitudes in Ishinomaki led to higher evacuation tendencies compared to that in Kamaishi. The box plots consist of the median line, box limits (inter-quartile range), whiskers (1.5 $\times$ inter-quartile range from upper or lower quartiles) and dots (fliers larger or smaller than whiskers). Each simulation case includes $n=120$ simulation runs.

We investigated the function of the urban structure under different evacuation tendencies by including leading evacuee agents who maintain a high positive evacuation attitude ($A_i=0.7$) in simulations (see Methods for details). Figure 4a shows the evacuation completion ratios with different numbers of leading evacuees. Overall, even a small number of leading evacuees has positive cascading effects and can improve evacuation tendencies in both cities, but the effect is more significant in Ishinomaki. The evacuation completion ratio in Ishinomaki was drastically improved with only 2% leading evacuees, i.e., from 4.7 to 43.0% on average. With a further increase in leading evacuees, the evacuation tendency in Ishinomaki surpassed that in Kamaishi by $\sim$ 7%. We analysed the ratio of the behaviour change caused by negative attitudes, i.e., the ratio of the agents who originally had a high attitude ($A_i \ge 0.5$) to evacuate but did not evacuate, and found that this significant improvement in Ishinomaki compared to Kamaishi corresponds to a low probability of the evacuating agents being influenced by clusters with negative attitudes (Fig. 4b), which was the main cause of the lower evacuation tendency in Ishinomaki in the simulations without leading evacuees. The dense grid-like urban structure in Ishinomaki reinforced negative evacuation attitudes under lower evacuation tendency; however, this feature with leading evacuees conversely becomes advantageous in promoting evacuations since the dense structure helps to effectively propagate the positive effects from leading evacuees.

## Discussion

With the agent-based evacuation model, we found that urban structures reinforce attitudes towards tsunami evacuation and largely affect the success of evacuation. Unlike the conventional tsunami evacuation models^38–42^ that have simplified decision-making processes including communications among evacuees, the model employed in this study can simulate comprehensive evacuation processes from decision-making with communications to detailed physical evacuation movements. This feature enables us to investigate the evacuation behaviours from attitudes towards evacuation. Consequently, the contribution of urban structure to evacuations including attitude formations could be evaluated while controlling the attitudes towards evacuations.

Our results provide a new explanation to the regional variation of the number of casualties in the 2011 Tohoku tsunami. Various factors such as individual and regional characteristics affect evacuation behaviours and cause complexity in observed behaviours during tsunamis^27^, which makes it difficult to evaluate the contribution of certain factors to the observed evacuation behaviours based only on fact-finding surveys. This complexity has led researchers to reach a slightly simple conclusion that the regional variations in the number of casualties in the 2011 tsunami can be explained by different levels of tsunami awareness among the local populations based on past tsunami experiences^43^. Our simulation results show that the distinctive urban structure found on the ria coast could have enhanced higher tsunami awareness in Sanriku coast while the grid-like urban structure often found in Sendai plain could reinforce lower tsunami awareness caused by less tsunami experience. This attitude reinforcement mechanism inherent in urban structures and different levels of regional tsunami awareness could have caused the observed significant difference in the number of tsunami casualties in the 2011 event. Since this paper focuses on showing the effect of urban structures on evacuation behaviours through controlled numerical experiments, we assumed uniform evacuation attitudes and ideal evacuation movements in the simulations. In reality, some trends of evacuation attitudes should exist, and people exhibit complex evacuation processes^26,44^. Further investigations of various evacuation scenarios will deepen the understanding of real evacuation behaviours.

The uncovered function of urban structures has paved the way for designing successful tsunami evacuations. Numerical simulations with leading evacuees have demonstrated that we can use urban structures to propagate positive evacuation attitudes and to drastically improve the evacuation tendency. These simulations have also revealed the potential that, with the attitude reinforcement mechanism of urban structures, only a small number of leading evacuees are required for the drastic improvement in evacuations. Conventional tsunami evacuation preparedness programmes aim to raise the overall level of tsunami awareness rather than training certain people, and it has been challenging. The simulations suggest that, along with these efforts, training some people to assume the role of leading evacuees is feasible and is an effective way to improve evacuations combined with the attitude reinforcement mechanism inherent in urban structures. The recently enhanced real-time detailed tsunami forecasting^8,11,12^ tailored for such trained people who can correctly interpret rich hazard information can support their decision-making and actions. Implementing a successful evacuation towards zero casualty is a challenge that has been referred to as a miracle; however, our results suggest that we can inevitably achieve evacuation success—the ‘miracle’—through harmonised urban and evacuation plannings that include evacuation route designs and optimal leading evacuee assignments, considering attitude reinforcement mechanisms. This study focuses on the effect of different urban structures on non-physical interactions among evacuees but lacks the point of view of physical evacuation efficiency. Actual tsunami evacuation often includes vehicular evacuation^45,46^, which can exhibit a significant difference in evacuation efficiency under different urban structures. In parallel with the aspect presented in this study, investigation of such physical evacuation efficiency will be essential for actual planning. The novel evacuation measure that uses the potential of urban structures with deliberate evacuation planning can be a clue to significantly encourage evacuation behaviours during natural disasters.

Although the importance of urban designs in improving physical evacuation efficiency, such as accessibility to safe places and transportation capacities^47,48^, has been recognised, non-physical aspects, such as communications and social cues occurred within populations in these environments, and their effect on evacuations have thus been far overlooked. This study, to the best of our knowledge, is the first to highlight the role of urban structures in controlling non-physical interactions among local populations and determining evacuation tendencies in natural disasters. Rapid global urbanisation is making urban areas denser and making people more vulnerable to natural disasters, especially with the impact of the recent climate change^49^. In addition to this known risk, the results of this study suggest that such urbanisation can have unexpected negative effects on evacuation behaviours, resulting in increased risks during natural hazards. Since the attitude reinforcement mechanisms revealed in this study depend largely on the direction of evacuation flows, even for the same urban configuration, various effects of the urban structure on evacuation behaviours are expected for different hazards. Hence, further investigations with various urban configurations and evacuation scenarios are essential to reveal and address potential risks. This study is the first step towards this effort but has opened this new research field of deep human-environment interactions in natural disasters.

## Methods

### Geospatial modelling

Geospatial models for Ishinomaki and Kamaishi cities were prepared based on public datasets. Based on existing studies^50,51^, the urban area on the right bank of Kyu-Kitakami River and the urban area on the left bank of Katsushi River were set as simulation domains for Ishinomaki and Kamaishi cities, respectively. For Ishinomaki city, the model was built using the latest geospatial data (road edge, building shapes and coastal lines) in 2011 before the 2011 Tohoku tsunami. These data were obtained from the Fundamental Geospatial Data (FGD), provided by Geospatial Information Authority of Japan (GSI)^52^. The FGD before the 2011 event is not available for Kamaishi city; thus, we synthesised the data using the 2012 FGD^52^ and damage survey data for the 2011 tsunami^53^. Some buildings which had been washed away and thus were not in the 2012 FGD, were filled using the damage survey data of 2011. Consequently, the urban structures of both cities at that time were reproduced with sufficient quality to perform analyses.

Using the constructed geospatial models, we calculated the navigation maps for agents. With a spatial cost map, the navigation maps were obtained as the minimum cumulative cost from destinations^45^. The cost map used in this study was created by assigning different cost values to different land uses (road: 1, building: 4000, water area: 8000, others: 2000). The resolution of the map was $1 \times 1~\textrm{m}^2$, sufficient to resolve the structure of the cities including narrow streets. The destination points were considered based on the following two criteria: (1) the points were located sufficiently far from the inundation area to ensure the safety of evacuees; (2) the points were the centres of roads connecting to areas distant from the inundation area. These criteria ensured that evacuees reached safe areas and were not isolated after inundation. All destination points that meet the criteria were considered to avoid an arbitrary choice. The number of destination points in Ishinomaki and Kamaishi are 5 and 15, respectively (Fig. 1b,c). With the calculated navigation map, evacuees prefer the shortest path towards the nearest destinations once they start to evacuate. It is known that real tsunami evacuation processes can become complex for family unification and safety or damage confirmation^44^; however, in the case of on-foot evacuation, the assumption is reasonable to represent general evacuation behaviour since the evacuees with limited mobility do not have sufficient time to exhibit complex movements during a short lead time before tsunami arrivals. The navigation maps were calculated using *r.cost* function in GRASS GIS^54^, which can be used from QGIS software^35^.

A total of 5000 agents were consistently considered both in Ishinomaki and Kamaishi models for comparison. Since previous studies^50,51^ conducted in areas that are almost equivalent to the simulation domain in this study estimated the population within the area as approximately 4423 and 4700 for Ishinomaki and Kamaishi, respectively, based on census data, the scale of the population is considered reasonable. The points were randomly generated within buildings in the simulation domain and considered as the initial positions for agents. To minimise unrealistic evacuation movements towards the coastline, only the buildings in the inundation area were used for generating initial positions in the Ishinomaki model. The random initial points were generated using *random points inside polygons* function available in QGIS^35^.

### Agent-based evacuation simulation

We used an agent-based on-foot tsunami evacuation model^30^, which simulates sequential evacuation processes of evacuees, i.e., the formation of the communication networks, communications to update their attitudes towards evacuation, decision-making and physical evacuation movements. In this model, agents have their own positive/negative attitudes towards evacuation as a psychological factor behind their behaviours, similar to evacuation intention^55,56^, i.e., positive/negative attitudes express the degree of intention to evacuate or not rather than a binary state. This model can be considered a simplified numerical implementation of the theoretical decision-making models^31–33^, which are often used to explain evacuation behaviours.

As people get verbal or non-verbal information (e.g., communicating with peers and seeing other people evacuating) for their evacuation decision-making in real tsunami events^26,27^, agents can update their evacuation attitudes through communications with surrounding agents. Such effect of surrounding people on evacuation decision-making is also empirically confirmed in other hazard events such as hurricanes^57^ and is an integral part of the well-known theoretical decision-making model^32,33^. The communication model is based on opinion dynamics models^58–60^. The attitude of agent *i* towards evacuation is represented as positive or negative values ($-1.0 \le A_i \le 1.0$) and updated through communications as in the following Eq. (1).

1 $$\begin{aligned} A_{i}(t_s + 1) = A_{i}(t_s) + {\mu } \frac{\sum _{j}I_{\epsilon }(A_{i}(t_s), A_{j}(t_s))(A_{j}(t_s) - A_{i}(t_s))}{\sum _{j}I_{\epsilon }(A_{i}(t_s), A_{j}(t_s))} \end{aligned}$$

Here $A_j$ are the attitudes of surrounding agents within a radius of $15~\textrm{m}$, $t_s$ is the time step for the communication model independent of the time in physical evacuation simulation, and $\mu$ is a parameter controlling attitudes’ update. $I_{\epsilon }$ is the indicator function expressed as in the following Eq. (2).

2 $$\begin{aligned} I_{\varepsilon }(A_{i}(t_s), A_{j}(t_s)) = {\left\{ \begin{array}{ll} 1 & |A_{i}(t_s) - A_{j}(t_s)| < {\varepsilon } \\ 0 & \text {otherwise} \end{array}\right. } \end{aligned}$$

This function allows interactions if and only if the difference between attitudes is within the bounded confidence $\varepsilon$ and thus can represent communications with cognitive biases^61^. For simulating evacuation behaviours, we employed the attitude-dependent bounded confidence presented in Ref.^30^. The initial attitudes of agents were given from a uniform random distribution. The leading evacuees were modelled as agents who have constant positive attitudes ($A_i=0.7$) that cannot be affected by any communications and can encourage surrounding agents to have higher attitudes. The same model parameters, including leading evacuee modelling, verified in Ref.^30^, were used for simulations.

When the attitude of an agent becomes rather positive ($A_i \ge 0.5$), the agent initiates evacuation movement towards the destination. Note that the communications always occur during the agents’ movements; agents stop evacuating when their attitudes become lower than the threshold. This modelling is based on the survey^44^, which reported people who initially intended to evacuate but later changed their behaviours based on their up-to-date information and attitudes. The physical evacuation movements were simulated using a microscopic agent-based tsunami evacuation model^62^, which is based on force-based crowd simulation models^63,64^. The movement of agent *i* is simulated by the following Eq. (3).

3 $$\begin{aligned} \frac{\textrm{d}{\textbf{v}}_{i}}{\textrm{d}t} = \frac{v^{0}_{i}\textbf{e}^{0}_{i} - \textbf{v}_{i}}{\tau _{\alpha }} + \sum _{i{\ne }j}\textbf{F}_{ij} \end{aligned}$$

where $\textbf{v}_{i}$ is the *i-*th current velocity, $v^{0}_{i}\textbf{e}^{0}_{i}$ is the *i-*th desired velocity, and $\tau _{\alpha }$ is the constant. The direction of the desired velocity is given from navigation maps generated from geospatial data. $\textbf{F}_{ij}$ is the interaction force among agents and is calculated by the formulation proposed in Ref.^64^. The interaction force is calculated based on the projected time to potential collisions between agents. All parameters were set as in Ref.^62^ since the model had been verified against real-world crowd movements.

### Stochastic simulation

Since stochastic components (initial attitudes and leading evacuee assignments in this study) in evacuation simulation can lead to different simulation results, stochastic simulations were conducted for evaluations. For simulations without leading evacuees, 120 simulation runs were executed for Ishinomaki and Kamaishi, respectively, changing the initial evacuation attitudes. Agents have different initial evacuation attitudes in different simulation runs, while overall attitudes always follow a uniform distribution. For simulations with leading evacuees, initial attitudes were fixed as in the median simulation cases, and the leading evacuee assignment was changed based on random sampling without replacement. We prepared 120 simulation runs for each setting to evaluate the effect of leading evacuees. The number of leading evacuees was carefully controlled so that the simulations in each case had the same number of leading evacuees (e.g., exactly 500 leading evacuees were considered for all simulation runs in the 10% case). We confirmed that the number of simulation runs is sufficient to capture the distribution of the results.

### Communication network analysis

Through the interactions among agents, communication networks, which become directed graphs, are formed in evacuation simulations. We extracted the strongly connected component (agents) in the communication networks as clusters for analyses, i.e., individual agents within a cluster can reach each other. We used NetworkX^65^ to analyse communication networks generated in evacuation simulations. The clusters of evacuees were extracted by applying *strongly_connected_components* function in NetworkX to the communication networks.

## Data Availability

The geospatial information used to build the urban structure was obtained from the Fundamental Geospatial Data^52^ provided by Geospatial Information Authority of Japan, which is publicly available (https://www.gsi.go.jp/kiban/). The damaged building data and the surveyed inundation extent used in model building and visualisation were obtained from Digital Archiving of the Great East Japan Earthquake Survey^53^ and are publicly available (http://fukkou.csis.u-tokyo.ac.jp/). The digital elevation model used for visualisation is publicly available from the Fundamental Geospatial Data^52^ (https://www.gsi.go.jp/kiban/), which is provided by Geospatial Information Authority of Japan.
